# Review on Carbonation Study of Reinforcement Concrete Incorporating with Bacteria as Self-Healing Approach

**DOI:** 10.3390/ma15165543

**Published:** 2022-08-12

**Authors:** Honin Ali Yahya Alshaeer, J. M. Irwan, Abdullah Faisal Alshalif, Amin Al-Fakih, Dina Yehia Zakaria Ewais, Abdelatif Salmi, Abdulmajeed Ali Alhokabi

**Affiliations:** 1 Jamilus Research Centre for Sustainable Construction (JRC-SC), Faculty of Civil Engineering and Built Environment, Universiti Tun Hussein Onn Malaysia, Parit Raja 86400, Johor, Malaysia; 2 Interdisciplinary Research Center for Construction and Building Materials, King Fahd University of Petroleum and Minerals, Dhahran 31261, Saudi Arabia; 3 Structural Engineering, Faculty of Engineering and Technology, Future University in Egypt, New Cairo 11835, Egypt; 4 Department of Civil Engineering, College of Engineering, Prince Sattam Bin Abdulaziz University, Alkharj 16273, Saudi Arabia; 5 Department of Civil Engineering, College of Engineering, Universiti Malaysia Pahang, Lebuhraya Tun Razak, Kuantan 26300, Pahang, Malaysia

**Keywords:** concrete, carbonation, bacteria, bio-concrete, self-healing

## Abstract

This study carried out a comprehensive review to determine the carbonation process that causes the most deterioration and destruction of concrete. The carbonation mechanism involved using carbon dioxide (CO_2_) to penetrate the concrete pore system into the atmosphere and reduce the alkalinity by decreasing the pH level around the reinforcement and initiation of the corrosion process. The use of bacteria in the concrete was to increase the pH of the concrete by producing urease enzyme. This technique may help to maintain concrete alkalinity in high levels, even when the carbonation process occurs, because the CO_2_ accelerates to the concrete and then converts directly to calcium carbonate, CaCO_3_. Consequently, the self-healing of the cracks and the pores occurred as a result of the carbonation process and bacteria enzyme reaction. As a result of these reactions, the concrete steel is protected, and the concrete properties and durability may improve. However, there are several factors that control carbonation which have been grouped into internal and external factors. Many studies on carbonation have been carried out to explore the effect of bacteria to improve durability and concrete strength. However, an in-depth literature review revealed that the use of bacteria as a self-healing mechanism can still be improved upon. This review aimed to highlight and discuss the possibility of applying bacteria in concrete to improve reinforcement concrete.

## 1. Introduction

The carbonation of concrete occurs when carbon dioxide from the air passes through the pores of the concrete and combines with calcium hydroxide to produce calcium carbonates [1]. The action of CO_2_ causes Ca (OH)_2_ to be converted into CaCO_3_, resulting in a minor shrinkage [2,3]. It can be seen clearly that carbonation cannot occur unless the CO_2_ reacts with another element. CO_2_ is converted to dilute carbonic acid in the presence of moisture, which destroys concrete and lowers its alkalinity [4]. The concentration of carbon dioxide (CO_2_) in the atmosphere has been steadily increasing from 280 parts per million (ppm) in preindustrial times to 381 ppm today, and some models predict that it will double within the next century. This increase in CO_2_ concentration has been caused by human activity [5]. The increase of carbonation caused by this change in the alkali level of the cement can be lower than the normal range, ranging from 12.5 to 13.5 of pH in normal concrete [6]. The high alkalinity safeguards the reinforcement bars from oxygen and water by forming a thin passivating coating around it. It will not corrode as long as it is kept in a very alkaline environment, which is called passivation. In practice, CO_2_ existing in the atmosphere in varying concentrations enters the concrete, carbonates the CO_2_, and decreases the alkalinity of the concrete. The pH of the pore water in the cured cement paste will be decreased from roughly 13 to around 9.0 [7]. When all of the Ca (OH)_2_ has been carbonated, the pH will drop to around 8.3. At such a low pH, the protective layer is removed, exposing the steel to corrosion [8]. One of the primary causes of reinforcing corrosion is the carbonation of concrete. Moreover, oxygen and moisture are also essential for embedded steel corrosion.

According to the previous study that was done by [9], the factors that affect the carbonation process have been categorized into external and internal factors. External factors that affect carbonation are the CO_2_ concentration, air pressure, relative humidity, and temperature. Internal factors, on the other hand, depend on physical characteristics such as the material size, surface area, and porosity of the concrete, or chemical properties such as the solid composition, calcium (Ca) contact, the Ca/Si ratio, the pH of the solid, and the availability of Pb, Cd, and Ni. The presence of CO_2_ in the environment surrounding the concrete is a significant factor that accelerates the carbonation process. To address these obstacles, self-healing approaches have been developed for improved outcomes. It is possible to improve the durability, compressive strength, and permeability of concrete. The ability to mend and seal concrete cracks was noted, and observations were made on the concrete’s permeability and its ability to mend cracks under pH-maintaining circumstances.

Bio-concrete is a self-healing technique that fills the cracks and pores in concrete with bacteria by inducing CaCO_3_ precipitation through bio-mineralization [10]. The acceleration of carbonation in bio-concrete is possible if the bacteria used can produce the carbonic anhydrase (CA) enzyme responsible for CO_2_ sequestration in the form of CaCO_3_ [11]. The mineral precipitation induced from bacteria has been identified as a technique for concrete crack healing that is environmentally friendly [12]. In this technique, the bacteria produce the urease enzyme to catalyze the hydrolysis of urea to CO_2_ and ammonia [13].

Microorganism-induced healing of concrete can be done by three various methods: by adding bacteria directly to the mix, by encasing bacteria in the mix to hold them inanimate until they are needed, or by spraying or injecting bacteria on the surface of the cracks [14]. In each of these cases, in addition to the bacteria, an organic food source, such as yeast extract [15] or a urea medium [16], has to be supplied in close proximity to them as needed so to guarantee that the surviving cells have the ability to potentially expand and react. In addition, there are two primary forms of CaCO_3_ precipitation: autotrophic and heterotrophic, both of which exhibit calcification and biomineralization [17]. When it comes to producing CaCO_3_ in concrete, only certain strains of alkaliphilic-based bacteria can be used [17,18]. This means that the pH of the concrete can be modified directly by the factors used to control the application of bacterial precipitation. 

This paper aimed to conduct a review on the role of bacteria and the self-heling process of carbonation on reinforcement concrete. At the same time, it reviews the mechanisms of the carbonation process, the factors affecting carbonation, and the effects of carbonation on concrete. In addition, the carbonation of concrete has a strong relation with the environment due to the role CO_2_ in this process [9].

## 2. Bibliometric Analysis by Co-Occurrence (Author Keywords)

Bibliometric analysis was used as scientific metrics to give a strong indicator and highlight the research gap in this review. Bibliometric maps were analyzed for 282 articles from the Scopus database using 5 different categories of limitations, as follows: keywords, last 5 years, type of documents (reviews and journal papers), English language. The keywords and their synonyms used were (“concrete”) AND (“self-healing” OR “self-healing”) AND (“carbonation” OR “CO_2_” OR “carbon dioxide”), respectively. The number of author keywords that appeared three times, the total link strength, and clusters were 50, 288, and 8, respectively. The author’s keywords that occurred more than 5 times were 22, as shown in Table 1. It can be seen that self-healing occurred more than 100 times with a high level of total strength in the selected articles. However, carbonation and corrosion protection occurred 6 times only, with a total strength of 9 and 5, respectively. This finding can give an indicator that the study of self-healing by previous researchers did not focus more on corrosion protection as well as the carbonation of concrete.

**Figure 1:**
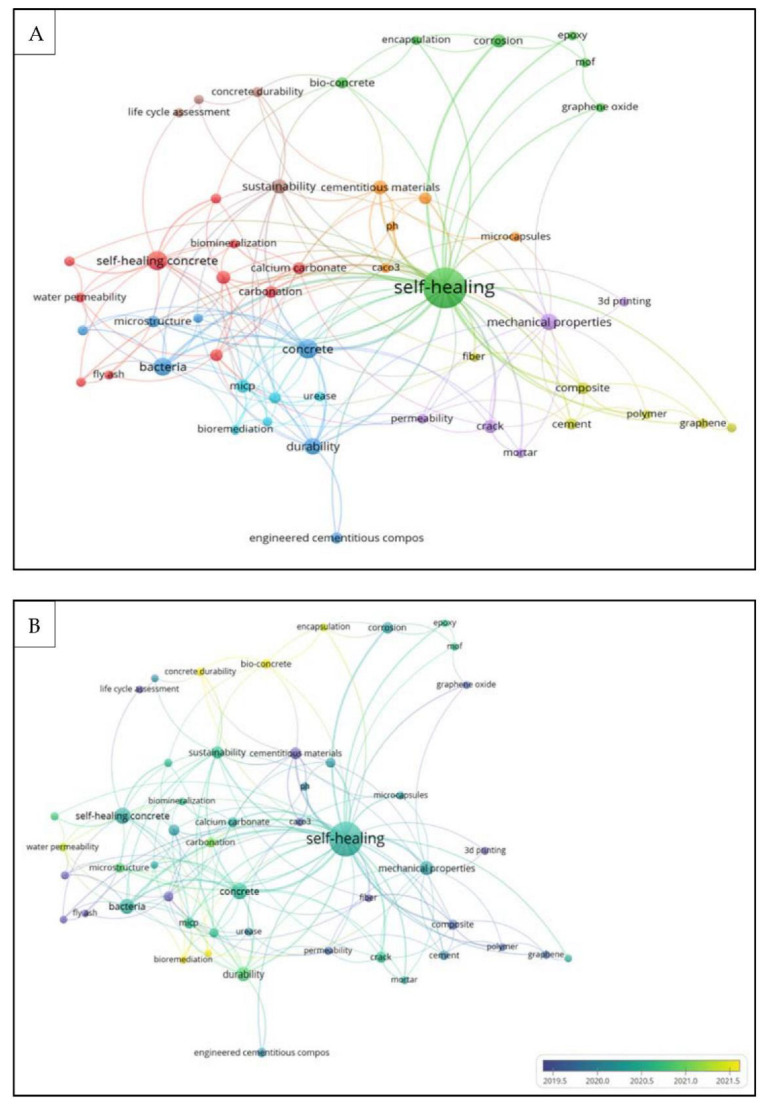
Bibliometric analysis of the keywords in publications of concrete self-healing and carbonation. (**A**) Network visualizations to show the frequency of keywords used by authors and the number of clusters; (**B**) Overlay visualizations to show the frequency of keywords used by authors colored by year and color.

The network visualization in Figure 1A represents the items by their label and circle. The size of the circles around the mentioned top 5 keywords is determined by the weight of the item. The large circle represents the higher weight of the items. The frequency of the keywords that occurred more than 5 times, and the weight of the items, indicated that most researchers worldwide are studying the new applications of self-healing in bio-concrete. This finding can give a clear view of the future applications of the corrosion protection of concrete reinforcement using a self-healing approach, which can be considered as the most applied in the last 5 years, as presented in overlay visualization map in Figure 1B.

## 3. Bibliographic Coupling Analysis (Countries)

**Figure 2:**
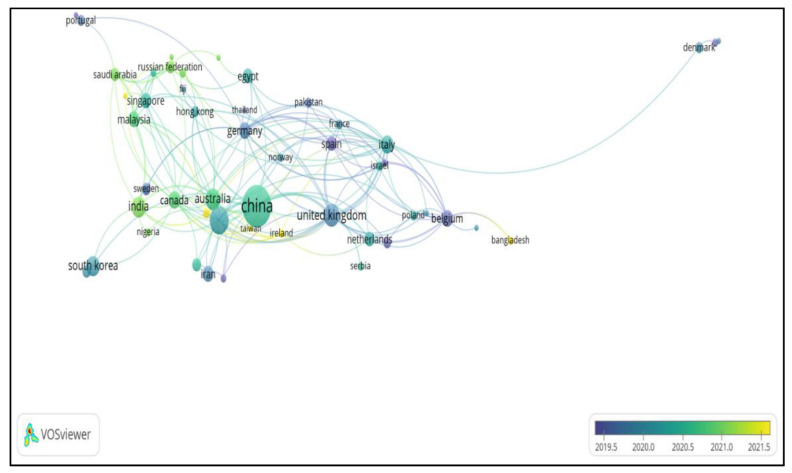
Bibliometric analysis of co-authorship (countries) Overlay visualizations occurrence of countries published, which are colored according to publication years of concrete self-healing and carbonation.

**Figure 3:**
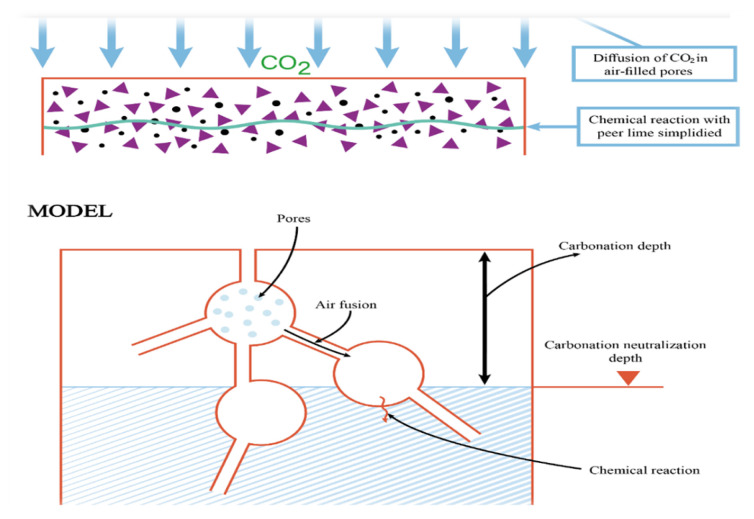
The mechanism of carbonation in reinforced concrete.

The bibliographic coupling analysis of the countries presents the total number of countries published in this research area. Only 51 countries have contributed according to research and more in this analysis. The countries that participated in the publications in this area are presented in Figure 2. The top five countries that published articles in this area are China, the United States, the United Kingdom, Australia, and India with 88, 32, 18, and 17, respectively. Unfortunately, most of these countries, such as the United States, the United Kingdom, Germany, and Belgium, raised this issue before and after 2019. as present in the overlay visualization map in Figure 3. However, countries such as China, Australia, India, Canada, and Malaysia have been focused on this area of research in 2020 and beyond. In addition, some countries have started to conduct some research in this regard, starting from 2021 up to now, such as Ireland, Taiwan, Cyprus, and Bangladesh. According to the previous discussion, it can be expected that this topic will have many applications in the coming years, as well as many publications worldwide.

## 4. Carbonation in Concrete

Carbonation can be classified into two types. The first is called weathering carbonation or natural carbonation, and the second type is called active carbonation or accelerated carbonation [19]. The weathering carbonation of concrete is considered as a passive carbonation [19,20]. According to [21], carbonation curing is different from weathering carbonation, in that the former takes place in a short period of time after casting, while the latter is a slow process afflicting mature concrete in service. Past research studied the effect of atmospheric weathering carbonation on the physical and transport properties of concrete [22]. The primary consequence of atmospheric carbonation is shrinkage, which can lead to unstable dimensional changes and cracking in the restraint [23]. Through active carbonation, the properties of the concrete can be maximized by gaining a higher early strength and a strong surface hardness, thereby reducing the porosity and enhancing the durability of the concrete [24]. In short, passive carbonation is the deterioration phase of concrete, whereas active carbonation is the beneficial phase of the concrete [25]. This beneficial manner of active carbonation was found to be feasible and practical for concrete without reinforced steel bars, such as concrete blocks, paving blocks, etc., and can be used as an alternative to the conventional accelerated steam-curing process [19]. The internal and external factors determine the rate of the passive carbonation process. The interior factors comprise the cement content, the strength of the concrete, the water-to-cement ratio, the porosity, and the degree of saturation in the concrete pore structure. This is in addition to the physical and chemical properties of the concrete materials, while the exterior parameters consists of the surrounding temperature, relative humidity, and CO_2_ concentration in the atmosphere [20]. 

As shown in Equation (1), carbonation in concrete is conducted by the chemical reaction between CO_2_ and Ca(OH)_2_ to form CaCO_3_ with the availability of water. As can be observed in Equation (2) [26], the C-S-H is likewise prone to decalcification when CO_2_ is present, which results in the production of CaCO_3_ and silica gel. Due to that, the pH of the concrete starts to decrease, resulting in the destruction of the passivation coating and causing the steel reinforcement to corrode over the long term [27].

Ca (OH)_2_ + CO_2_ → CaCO_3_ + H_2_O (1)

C-S-H + 2CO_2_ → SiO_2_ + 2CaCO_3_ + H_2_O (2)

According to Abdul-Baki [27], early carbonation curing is a very fast process that occurs within hours of casting. As indicated in Equations (3) and (4), most of the cement paste’s anhydrous components (C2S and C3S) are involved in the reaction, which would otherwise be engaged by water.

C3S + (3 − x) CO_2_ + yH_2_O → CxSHy + (3 − x) CaCO_3_ (3)

C2S + (2 − x) CO_2_ + yH_2_O → CxSHy + (2 − x) CaCO_3_ (4)

As a carbon sink, early-age carbonation curing is a great advantage in the concrete industry as well [28]. Climate change can be mitigated by using carbonation curing, which converts carbon dioxide into stable, leak-proof carbonates. By causing calcium carbonate precipitation through biomineralization, bio-concrete can be used to fix concrete fissures [29]. In this case, water pours out of the fissures in the concrete and into the concrete’s capillary pores. Bacterial spores will germinate in the presence of water and nutrients, and the limestone will form on top of the crack as a result. This will help close it up and prevent further damage. Aside from the oxidation of organic acids and the hydrolysis of urea, the most frequent microorganisms utilized to fill fractures in the concrete or mortar by making limestone are nitrate-reducing bacteria [30].

## 5. Factors Influence the Kinetics of the Carbonation Reaction in Concrete

Numerous variables have an impact on the rate at which carbon dioxide is extracted from concrete. They have been divided into internal and external aspects as shown in Table 2, which are how people see things. Studying carbonation was done to counteract the deteriorating mechanism of weathering carbonation on hydrated concrete.

## 6. Carbonation Process in Concrete

Calcium carbonate (CaCO_3_) is formed through the carbonation process on the surface layer of cement-based materials. The CaCO_3_ generated is then deposited into the pore network through the hydrated cement matrix, causing a refinement in the carbonated layer’s pores [51]. According to Liisma, Sein, and Järvpõld [52], the carbonation process is most active in the relative humidity (RH) range from 50 to 70%.

In the presence of moisture, it can form

(5) $${\mathrm{CO}}_{2}+H_{2}O\to H_{2}{\mathrm{CO}}_{3}$$

This carbon acid can react with sodium hydroxide:

(6) $$H_{2}{\mathrm{CO}}_{3}+\mathrm{NaOH}\;\to {\;\mathrm{Na}}_{2}{\mathrm{CO}}_{3}+H_{2}O$$

This sodium carbonate or potassium carbonate can react with calcium hydroxide in the cement to form calcium carbonate and gain water:

Na_2_CO_3_ + Ca (OH)_2_ = CaCO_3_ + 2NaOH (7)

K_2_CO_2_ + Ca (OH)_2_ = CaCO_3_ + k_2_OH (8)

The carbonated zone causes a chemical reaction to occur with the hydrated minerals in the concrete and decreases the alkalinity. Since carbonation penetration into the concrete is influenced by changes in the pH and CO_2_ content [4], a continuous process of CO_2_ absorption by the hardened concrete results in a decrease in the alkalinity of the concrete (pH < 9), thus making its reinforcement vulnerable to corrosion and leading to crack formation [53]. This negative aspect of passive carbonation is described as the deterioration mechanism of reinforced concrete [19]. 

In this process, calcium hydroxide will get dissolved more into the pour solution. After that, the alkaline materials including sodium hydroxide and potassium hydroxide will be consumed by carbon dioxide in the presence of moisture-forming carbonate. Carbonation-induced corrosion of rebars occurs as a result of the disintegration of the passive surface layer [54]. Boualleg et al. [55] revealed that substantial CO_2_ penetration causes insoluble CaCO_3_ to be converted to soluble Ca(HCO_3_)_2_. The Ca(HCO_3_)_2_ is easily leached off, thereby resulting in the lowering of the porosity of the hydrated cement [13]. Carbon dioxides penetrate through the pore system and the moisture present causes carbonation on the surface, as seen in Figure 3. If there is no moisture, there will be no reaction because it occurs in the solutions of the carbonic acid, which was not formed. Therefore, carbon dioxide in water is necessary because the carbon dioxide concentration will be very low when dissolved in water. If it is a fully dry concrete reaction, carbon dioxide will be absent. It has also been noted that the reaction only occurs at specific humidities. When the carbonation shrinkage was around 55%, the humidity was at its peak. Additionally, carbonation is at its most efficient around 50–60 RH; if it is entirely moist, no carbonation will occur [56,57]. This implies that carbonation only occurs when both oxygen and carbon dioxide are present. To increase the concrete strength and durability, blended cement, and supplementary cementitious materials such as fly ash, limestone, silica fume, etc., should be used [50].

## 7. Carbonation Curing Setup

**Figure 4:**
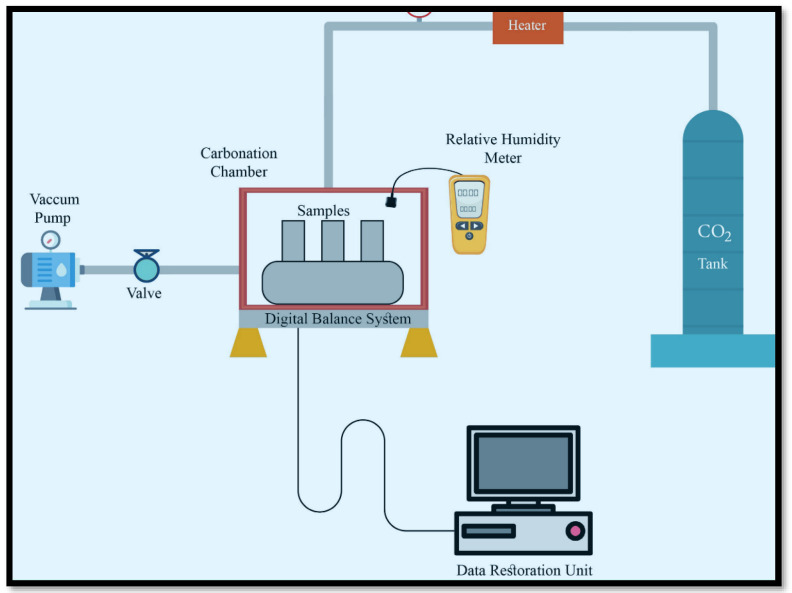
The carbonation process for concrete specimens in the chamber.

Carbonation curing refers to the process of exposing concrete to carbon dioxide gas for an extended period. CO_2_ is usually delivered into a closed chamber and left for a particular amount of time and under specific conditions for the reaction to occur. Many studies have used a static carbonation system, as shown in Figure 4 [58,59,60,61]. For a high carbonation degree, it was crucial to introduce initial air curing before the carbon exposure. This was followed by carbonation curing, which was done by injecting CO_2_ gas (99% purity) into a sealed chamber [62]. A gas regulator was used to control the chamber pressure for the required pressure level to be attained for a specific period [63,64]. The gas tank was fitted with a single-stage regulator to moderate the gas pressure from the tank to the pressure vessel. 

The pressure gauge for the tank ranged from 0 to 28 MPa with a 1 MPa precision, while the outlet gauge pressure range was from 0 to 1.4 MPa with a 0.05 MPa precision [60]. Regulators were adjusted to attain the desired pressure level and maintain it during the carbonation curing. The temperature of the carbon dioxide gas was lower than room temperature because it was in a highly compressed liquid/gas state in the cylinder [20]. As the carbon dioxide exited the tank, it was heated with an electric heater attached between the tank and regulator. The heater was manufactured by Matheson and controlled with a thermostat to prevent the overheating of the gas [65]. The pressure was reduced to 0.7 bars below the atmospheric pressure within a few seconds before carbonation through a vacuum [59,66]. As the carbon dioxide gas (99% purity) flowed into the sealed chamber, the heater installed at the inlet raised it to room temperature. A constant pressure of 0.1 MPa was retained while the pressure regulator was connected to the system. To monitor the increase in mass from the carbon reaction, the whole system was placed on a digital scale [59]. The carbonation chamber was used to carbonate the unmolded concrete sample for different periods (1, 2, 3, 6, 18, and 24 h) with a pressure level of 0.1 Bar [61]. For an increase in the CO_2_ sequestration, the samples were preconditioned in a 25 °C environmental chamber with a relative humidity of 50% for 6 h. Simultaneously, the air-cured specimens were put in the same laboratory conditions for 28 days [61]. Most studies on early-age carbonation curing were conducted using pure CO_2_ (99.5%) as opposed to flue gas (between 10–25% CO_2_ content) for reaction efficiencies [59,63]. A curing scheme with three stages has been proposed. Each step plays a unique role in enhancing the early and late performance of the concrete [20].

By using the setup in Figure 4, Abdul-baki [27] selected three separate carbonation curing regimes to test.

➢ Pressurized carbonation curing

**Figure 5:**
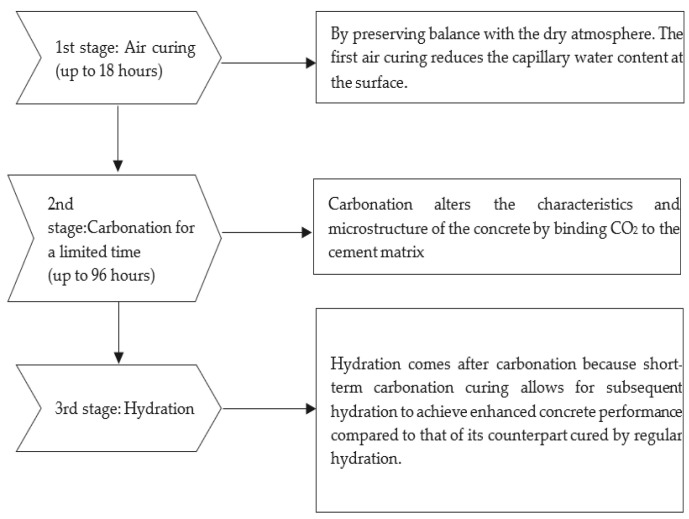
The carbonation of concrete at the earlier curing stages.

The CO_2_ gas was fed into the chamber without first vacuuming it for pressure-aided carbonation curing. The carbonation chamber was pressurized by keeping the valve open, as seen in Figure 5. The pressure was kept constant at 100 kPa (14.5 psi) during the curing process. This regime served as the benchmark for early carbonation curing previously adopted in past studies [55,56,57,58]. Consequently, the pressurized carbonation samples were used as a means of comparing the positive pressure carbonation curing to the ambient curing.

➢ Vacuum carbonation curing

A venturi pump, as shown in Figure 2, was linked to an air compressor inlet that functioned as a vacuum for carbonation curing. The carbonation chamber was sucked to the target subpressure once the valve was opened. The requisite negative vacuum pressures were achieved in the laboratory in under 5 min. As a result, aiming to reduce the vacuum and CO_2_ pressures was critical to a successful pilot-scale test.

## 8. Curing Procedures for Carbonation in Concrete

Certain researchers have studied the curing procedures of carbonation in different batches using rectangular slab samples [20,27]. The procedures have been summarized in Table 3.

For comparison, batches 1 to 4 are steam-cured, while batches 5 to 14 are carbonation-cured. Steam curing occurred in a steam cooker for 4 h, with a maximum temperature of 80 °C and relative humidity of 95% [27]. Initial curing of 0, 4, 6, and 8 h at 22 ± 1 °C and relative humidity of 80% was applied before the steam. Initial curing at 0 h was immediate for the carbonation of fresh concrete and was set as a reference. The initial curing of 18 h was carried out to simulate overnight curing and is possibly the longest preset accepted by commercial production. After the initial curing, the concrete slab samples were placed in a sealed chamber, as seen in Figure 5, vacuumed to approximately 0.7 bars below the atmosphere, and then filled with carbon dioxide gas to a pressure of 1 bar [59]. During carbonation, the mass curve of the concrete was obtained by placing the chamber on a digital balance. The duration of the carbonation ranged from 2 to 4 h. The effect of the extreme exposure time was studied over a period of 96 h [20]. 

The procedure for carbonation curing of paste and concrete samples have also been studied in the previous studies, and were broken into four steps [28,67].

Step 1: In-mold curing. Wet-mix concrete cannot be unmolded after casting because it has a higher slump. Therefore, it is important to carry out the initial hydration curing on the mold. The mixture proportions determine the time required for this step. A study by Zhang et al. [64] noted that approximately 5 h was needed to reach the initial set at ambient conditions (25 °C and 60% relative humidity) in the open air. It is also used to remove a part of the mixing water for carbonation.

Step 2: Off-mold preconditioning. After the first step, the cube samples were unmolded and left to fan dry on the bottom plates. This was done at a wind speed of 1 m/s for 5.5 h in a room of 25 °C and 50 ± 5% RH. This step is important because the conditioning removes more of the free water to allow for carbon dioxide penetration and the precipitation of carbonates.

Step 3. Carbonation curing. The cube samples were placed in a pressure chamber with Co2 gas (99.8% purity) for carbonation under a constant pressure of 5 bar for 2, 12, and 24 h.

Step 4. Subsequent hydration. Postcarbonation, the cubes were placed in a moisture room (25 °C, 95% RH) for 27 more days after hydration. Preparation was also made for the hydration references. The curing was carried out in a sealed mold within the first 24 h, then unmolded, and further cured in the same moisture room for 27 days.

## 9. Carbonation in Bio-Concrete

According to Farah et al. [68], carbonation is one of the major elements that might influence the longevity of concrete constructions. Concrete carbonation was commonly characterized as a chemical reaction that produces air carbon dioxide quantities and cement hydration components, especially as it relates to the reaction [69]. This phenomenon may influence the serviceability of the structures. In the last decade, bacteria calcium carbonate precipitation has been increasingly exploited to promote self-healing and to enhance the characteristics of concrete materials [70,71].

**Figure 6:**
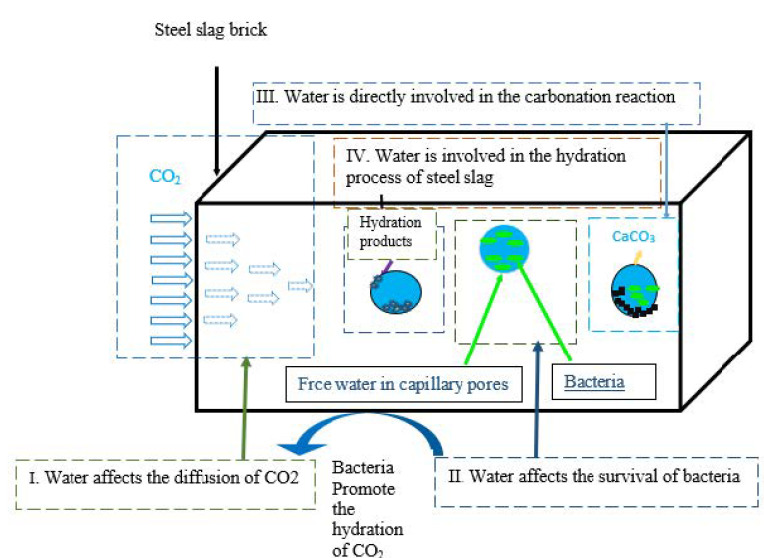
Effect of bacteria concentration on the carbonation process [82].

Numerous approaches have been utilized in recent years to improve the carbonation rate and reduce the preparation cycle of carbonated steel slag, such as raising the carbonation pressure [72,73], raising the carbonation temperature [74], decreasing the size of the steel slag particulate [75], and carbonating the steel slag into a film form and slurry [70,76]. However, the method of producing carbonated steel slag is difficult and expensive. The few investigations on the relationship of the carbonation processes with microorganisms that have been undertaken so far revealed that bacteria struggle to remain active in the extremely high alkalinity of the steel slag [67,77]. Furthermore, studies have discovered that introducing bacteria into the steel slag system aids in increasing the carbonation speed, enhancing the carbonation impact (within 2 h), and strengthening the carbonated product development [67,78]. Tittelboom et al. [79] examined how bacteria can be used to repair concrete cracks, and observed that pure bacterial cultures could not heal the cracks. However, it was discovered that using silica gel to protect the bacteria helped in filling the cracks. Similarly, Wiktor and Jonkers [80] noted that bacterial therapy could be used to close cracks with diameters as wide as 0.46 mm. Additionally, Luo et al. [47] observed that, 5 days after bacteria was used, most of the cracks (up to 0.3 mm) in the concrete healed, and it took 20 days to fill the surface. An alternative and eco-friendly technique suggested for the self-healing of cracks is bacteria-precipitated CaCO_3_ [81]. This technique was proposed by Jonkers et al. [74] after observing that bacteria-generated CaCO_3_ reduces the porosity and boosts the compressive strength. Similar reports were given by Vijay and Murmu [82] after discovering that adding bacteria to concrete increases the strength by using CaCO_3_ from the metabolic activity of bacteria to fill the pores, as seen in Figure 6. It was noted that injecting bacteria into the fractures in concrete pavement increased the samples’ resistance to freeze–thaw cycles compared to those with microorganisms [83]. Luo et al. [79] used an electron microscope to examine the bacterial deposits left on the surface of cement paste specimens. The study evaluated the influence of the crack width, processing method, and crack age on the ability of cracks in cement paste to self-repair. The findings revealed that the bacterial treatment was revealed to be capable of repairing cracks with a width of up to 0.8 mm [84] A study by Wang et al. [85] revealed that water influences the whole microbial carbonation process in four ways, as shown in Figure 6.

- Water content may affect carbon dioxide diffusion.
- Water content has a huge effect on bacterial survival, which may further affect CO_2_ hydration and calcium carbonate deposition rates.
- Water plays a major role in carbonation reactions.
- Water is involved in the hydration process of steel slag to assess the water impact on the carbonation reaction and the survival of bacteria, and the concept of ‘residual water–cement ratio’ is presented. This idea was originally developed to research the precuring procedure for concrete with CO_2_ [86].

## 10. The Role of Bacteria on Carbonation of Reinforce Concrete 

Bacteria have been used in the majority of studies involving self-healing concrete based on its ability to generate calcium carbonate sediments using urea and calcium supplies via the catalysis of urea hydrolysis into ammonia and carbonate to generate urease [87,88]. Bacteria induces the creation of minerals, which is a burgeoning field of study known as biomineralization [89]. These biogenic minerals produce a protective coating on the surface that resists the uptake of water and toxic components while also acting as a cementing layer. At the same time, bacteria produce the urease enzyme, which has the ability to increase the pH in the concrete. The increase of the pH in the concrete due to the urease enzyme production may maintain the high level of alkalinity of the concrete, even once the carbonation process has occurred. Therefore, this finding requires further study in labs to confirm the theory. In addition to that, microbially induced calcite precipitation (MICP) carbonatogenesis has received a lot of interest and has been recommended as an environmentally beneficial way for protecting damaged ornamental stones [90]. It has been widely researched for the development of construction materials by means of surface treatment, the increase of the compressive strength, reduced water absorption, chloride ion permeability, and crack remediation [85].

## 11. Conclusions

This paper discussed the self-healing of bacterial concrete as well as the effectiveness of the self-healing of fractures by encapsulated bacteria on the recovery of a variety of mechanical and durability qualities. The findings that can be drawn after a comprehensive investigation are that bacteria can contribute in protect steel in concrete via the self-healing process and maintain the level of the pH in concrete at a high level. However, there are many factors affecting the carbonation process, some which can be classified to two groups: internal factors and external factors. Therefore, bacteria alone cannot play a superior role if the role of these factors have been neglected. The bacteria can be considered as an additional material in the reinforced concrete if the bacteria used has the ability to produce the urease enzyme, which can help to in crease the pH of the concrete. In addition, the properties of the concrete improve especially the compressive strength and a reduction in the amount of water and chloride ion that can travel through a substance when bacteria are present.

## Figures and Tables

**Table 1:** The author’s keywords occurred more than 5 times in VOS viewer software.

Verify Selected Keyword			
Selected	Keyword	Occurrence	Total Link Strength
**✓**	Self-healing	104	83
**✓**	Concrete	17	30
**✓**	Bacteria	14	20
**✓**	Durability	12	19
**✓**	Sustainability	9	17
**✓**	Calcium carbonate	6	12
**✓**	Cementitious materials	8	11
**✓**	Mechanical properties	12	11
**✓**	Composite	6	10
**✓**	Bacillus subtitles	7	9
**✓**	Carbonation	6	9
**✓**	Crack	7	9
**✓**	Marine environment	5	8
**✓**	Microstructure	6	8
**✓**	Corrosion	7	7
**✓**	Micp	7	7
**✓**	Compressive strength	6	6
**✓**	Self-healing concrete	17	6
**✓**	Cement	5	5
**✓**	Corrosion protection	6	5
**✓**	Bio-concrete	5	4
**✓**	Engineered Cementitious composite (ecc)	5	4

**Table 2:** Carbonation in concrete is affected by both internal and external influences [31,32,33].

Internal Factors	Their Effect on Carbonation in Concrete	External Factors	Their Effect on Carbonation in Concrete
Porosity	An increase in the depth of carbonation shows that, when the amount of water in a substance goes up, so does its porosity. As a direct consequence of this, the level of carbonation that is present is elevated [34]. However, if the amount of water content drops, the porosity will also decrease, which will result in a shallower layer of carbonate.This is evidence that there is a linear relationship between carbonation and porosity, according to previous research done by [35].	Curing period	Concrete curing has a significant impact on many of the qualities of the material. Due to this, it enhances concrete strength while decreasing carbonation depth. As a result, it was determined that the curing period has a direct impact on the permeability, concrete strength, and carbonation depth [36].
Water/Cement ratio	The proportion of water to cement has a considerable bearing on the resulting carbonate [37], where the proportion of water to cement in a mixture is directly related to the carbonation depth. When there is a lower proportion of water to cement, the carbonation depth is significantly shallower, but it rises when there is a higher proportion of water to cement [38].	relative humidity	The carbonation process is worse in 50–70% of RH settings [4,39,40]. When relative humidity (RH) is above 70%, water fills the pores, preventing CO_2_ from entering cement-based structures. However, when the relative humidity falls below 50%, pores dry out, and Ca(OH) and CO_2_ do not dissolve for carbonation [4,41].
Grade of concrete	Carbonation is limited to the concrete’s surface layers, according to the previous studies. Concrete’s depth may only increase 20 mm in 50 years, whereas porous concrete’s carbonation can reach 100 mm. This is due to the fact that dense concrete is able to successfully resist the diffusion of carbon dioxide more efficiently than porous concrete [42].	Ca(OH)_2_ concentration	The amount of calcium hydroxide in cementitious materials affects how long they last against carbonation [1,43]. This is because calcium hydroxide has a high pH of 12.6. Since more total calcium hydroxide can hold more CO_2_, it is generally agreed that the amount of calcium hydroxide has a big effect on the rate of carbonation [3].
Depth of cover	It has been found that, when the correct plastering is used, carbonation and carbonate depth reduce significantly [44]. The effect of carbonation can be reduced by using a cover with a thickness up to 40 mm [45]. Carbon dioxide diffusion is slowed by protected surfaces, reducing the impact of carbonates.The recommended amount of carbonate protection is at least 25 mm [46].	Temperature	Chemical reactions are very sensitive to the temperature [47]. As the temperature goes up, the concrete’s compressive strength goes down. Even though the rate of carbonation in concrete is not linearly related to temperature, CO_2_ concentration in concrete is the most important factor, and CO_2_ transmission controls the whole process of carbonation [48].
Type of cement	Compared to regular Portland cement, the carbonation rate of blended cements has a greater value [31].Additionally, pozzolanic reactions in mixed cements make use of CH as a reactant. Because of this, there is less CH accessible, and carbonation occurs more quickly, in comparison to OPC, which has a greater quantity of CH [28].	Concentration of (CO_2_)	Rising CO_2_ levels reduce concrete’s compressive strength. This increases carbonation and affects aqueous cement strength [42]. High CO_2_ concentrations increase water content in pores due to quick reaction rate and water evolution [49]. When thin samples are examined at the correct RH, carbonates with low carbon dioxide concentrations develop faster [50].

**Table 3:** Curing procedures batch.

Batch	Condition	Initial Curing			Steam Curing			Carbonation Curing		Subsequent Hydration
		RH%	T °C	t (Hours)	RH %	T °C	t (Hours)	t (Hours)	Water Spray (g)	t (Days)
1	0 a + 4 s	-	-	0	95±	75 ± 5	4	-	-	28
2	4 a + 4 s	80 ± 5	22 ± 1	4	95±	75 ± 5	4	-	-	28
3	6 a + 4 s	80 ± 5	22 ± 1	6	95±	75 ± 5	4	-	-	28
4	8 a + 4 s	80 ± 5	22 ± 1	8	95±	75 ± 5	4	-	-	28
5	0 a + 4 c	-	-	0	-	-	-	4	-	28
6	0 a + 4 c ^w^	-	-	0	-	-	-	4	1 ± 0.2	28
7	4 a + 4 c	50 ± 1	25 ± 0.2	4	-	-	-	4	-	28
8	4 a + 4 c ^w^	50 ± 1	25 ± 0.2	4	-	-	-	4	17 ± 2	28
9	6 a + 4 c	50 ± 1	25 ± 0.2	6	-	-	-	4	-	28
10	8 a + 4 c	50 ± 1	25 ± 0.2	8	-	-	-	4	-	28
11	18 a + 4 c	50 ± 1	25 ± 0.2	18	-	-	-	4	-	28
12	18 a + 4 c ^w^	50 ± 1	25 ± 0.2	4	-	-	-	4	29 ± 2	28
13	18 a + 2 c	50 ± 1	25 ± 0.2	18	-	-	-	2	-	28
14	18 a + 96 c	50 ± 1	25 ± 0.2	18	-	-	-	96	-	28

Notice: a—Initial air curing; s—Steam curing; c—Carbonation; RH—Relative humidity; T—Temperature; t—Time. w—Water sprayed after carbonation [20,27].

## Data Availability

The results of the study are not placed in any publicly archived datasets.

## References

[1] Parameswaran L., Kumar R., Sahu G.K. (2008). Effect of Carbonation on Concrete Bridge Service Life. J. Bridge Eng..

[2] Zhao H., Sun W., Wu X., Gao B. (2018). The Effect of the Material Factors on the Concrete Resistance Against Carbonation. KSCE J. Civ. Eng..

[3] Kalhori H., Bagherpour R. (2017). Application of carbonate precipitating bacteria for improving properties and repairing cracks of shotcrete. Constr. Build. Mater..

[4] Alshalif A.F., Irwan J., Othman N., Zamer M., Anneza L. (2017). Carbon Dioxide (CO_2_) Sequestration in Bio-Concrete, An Overview. MATEC Web Conf..

[5] Mors R.M., Jonkers H.M. (2019). Bacteria-based self-healing concrete: Evaluation of full scale demonstrator projects. RILEM Tech. Lett..

[6] Navneet C., Anita R., Rafat S. (2011). Calcium carbonate precipitation by different bacterial strains. Afr. J. Biotechnol..

[7] Chi J.M., Huang R., Yang C.C. (2002). Effects of carbonation on mechanical properties and durability of concrete using accelerated testing method. J. Mar. Sci. Technol..

[8] Tayebani B., Mostofinejad D. (2019). Penetrability, Corrosion Potential, and Electrical Resistivity of Bacterial Concrete. J. Mater. Civ. Eng..

[9] Alshalif A.F., Irwan J.M., Othman N., Al-Gheethi A.A., Shamsudin S. (2020). A systematic review on bio-sequestration of carbon dioxide in bio-concrete systems: A future direction. Eur. J. Environ. Civ. Eng..

[10] Khattab I.M., Shekha H., Abdi M.A. (2019). Study on Self-healing Concrete types—A review. Sustain. Struct. Mater. Int. J..

[11] Xiao X., Goh L.X., Unluer C., Yang E.-H. (2021). Bacteria-induced internal carbonation of reactive magnesia cement. Constr. Build. Mater..

[12] Antiohos S., Papadakis V., Tsimas S. (2014). Rice husk ash (RHA) effectiveness in cement and concrete as a function of reactive silica and fineness. Cem. Concr. Res..

[13] Balam N.H., Mostofinejad D., Eftekhar M. (2017). Use of carbonate precipitating bacteria to reduce water absorption of aggregates. Constr. Build. Mater..

[14] Joshi S., Goyal S., Mukherjee A., Reddy M.S. (2017). Microbial healing of cracks in concrete: A review. J. Ind. Microbiol. Biotechnol..

[15] Jawaid S., Ahmed K., Bhutto M.A. (2018). Bio Concrete: An Overview. Int. J. Biol. Biotechnol..

[16] Talaiekhozani A., Abd Majid M.Z. (2014). A Review of Self-healing Concrete Research Development. J. Environ. Treat. Tech..

[17] Abdulaziz Al-Shalif A.F. (2020). Sequestration of Carbon Dioxide (CO_2_) Using Bio-Foamed Concrete Brick Incorporating Bacillus Tequilensis Bacteria. Ph.D. Thesis.

[18] Martirena F., Rodriguez-Rodriguez Y., Callico A., Diaz Y., Bracho G., Hereira A., De Leon J.G., Sorelli L., Alvarado-Capó Y. (2016). Microorganism-based bioplasticizer for cementitious materials. Biopolymers and Biotech Admixtures for Eco-Efficient Construction Materials.

[19] Kaliyavaradhan S.K., Ling T.-C. (2017). Potential of CO_2_ sequestration through construction and demolition (C&D) waste—An overview. J. CO_2_ Util..

[20] El-Hassan H. (2013). Static and Dynamic Carbonation of Lightweight Concrete Masonry Units.

[21] Simović T., Barjaktarović L. (2011). Securities portfolio optimization at the Belgrade stock markets on the basis of the Harry Markowitz’s theory. Singidunum Rev..

[22] Papadakis V.G., Vayenas C.G., Fardis M. (1991). Experimental investigation and mathematical modeling of the concrete carbonation problem. Chem. Eng. Sci..

[23] Seifan M., Samani A.K., Berenjian A. (2016). Bioconcrete: Next generation of self-healing concrete. Appl. Microbiol. Biotechnol..

[24] De Belie N. (2016). Application of bacteria in concrete: A critical evaluation of the current status. RILEM Tech. Lett..

[25] Waghmare P.A.P., Mulka A., Dhumal K., Kamal S., Warule S. (2018). Self-Healing Concrete or Bio- Concrete used in Construction Industry. IJSDR.

[26] Rostami V., Shao Y., Boyd A.J. (2011). Durability of concrete pipes subjected to combined steam and carbonation curing. Constr. Build. Mater..

[27] Abdul-baki G. (2017). Development of Vacuum Carbonation Curing Technology for Concrete at Ambient Conditions.

[28] Zhang D., Shao Y. (2016). Early age carbonation curing for precast reinforced concretes. Constr. Build. Mater..

[29] Griño J.A.A., Daly M.K.M., Ongpeng J.M.C. (2020). Bio-Influenced Self-Healing Mechanism in Concrete and Its Testing: A Review. Appl. Sci..

[30] Algaifi H.A.A., Abu Bakar S., Sam A.R.M., Abidin A.R.Z. (2018). Crack-healing in cementitious material to improve the durability of structures: Review. MATEC Web Conf..

[31] Marangu J.M., Thiong’o J.K., Wachira J.M. (2019). Review of carbonation resistance in hydrated cement based materials. J. Chem..

[32] Alshalif A.F., Irwan J.M., Tajarudin H.A., Othman N., Al-Gheethi A.A., Shamsudin S., Altowayti W.A.H., Sabah S.A. (2021). Factors Affecting Carbonation Depth in Foamed Concrete Bricks for Accelerate (CO_2_) Sequestration. Sustainability.

[33] Tan L., Reeksting B., Ferrandiz-Mas V., Heath A., Gebhard S., Paine K. (2020). Effect of carbonation on bacteria-based self-healing of cementitious composites. Constr. Build. Mater..

[34] Balam N.H., Mostofinejad D., Eftekhar M. (2017). Effects of bacterial remediation on compressive strength, water absorption, and chloride permeability of lightweight aggregate concrete. Constr. Build. Mater..

[35] Gonen T., Yazicioglu S. (2007). The influence of mineral admixtures on the short and long-term performance of concrete. Build. Environ..

[36] Rao N.V., Meena T. (2017). A review on carbonation study in concrete. IOP Conf. Ser. Mater. Sci. Eng..

[37] Chopra D., Siddique R., Kunal (2015). Strength, permeability and microstructure of self-compacting concrete containing rice husk ash. Biosyst. Eng..

[38] Siddique R., Jameel A., Singh M., Barnat-Hunek D., Kunal, Aït-Mokhtar A., Belarbi R., Rajor A. (2017). Effect of bacteria on strength, permeation characteristics and micro-structure of silica fume concrete. Constr. Build. Mater..

[39] Pravalika A., Rao N.V. (2018). Effect of carbonation on the properties of concrete. Int. J. Civ. Eng. Technol..

[40] Kumar J., Sathish Kumar K., Dayakar P. (2014). Effect of microsilica on high strength concrete. Int. J. Appl. Eng. Res..

[41] Rukzon S., Chindaprasirt P. (2010). Strength and Carbonation Model of Rice Husk Ash Cement. J. Mater. Civ. Eng..

[42] von Greve-Dierfeld S., Lothenbach B., Vollpracht A., Wu B., Huet B., Andrade C., De Belie N. (2020). Understanding the carbonation of concrete with supplementary cementitious materials: A critical review by RILEM TC 281-CCC. Mater. Struct..

[43] Han S.H., Jun Y., Shin T.Y., Kim J.H. (2020). CO_2_ Curing Efficiency for Cement Paste and Mortars Produced by a Low Water-to-Cement Ratio. Materials.

[44] Krishnapriya S., Babu D.V. (2015). Isolation and identification of bacteria to improve the strength of concrete. Microbiol. Res..

[45] Castellote M., Fernandez L., Andrade C., Alonso C. (2009). Chemical changes and phase analysis of OPC pastes carbonated at different CO_2_ concentrations. Mater. Struct..

[46] Chahal N., Siddique R., Rajor A. (2012). Influence of bacteria on the compressive strength, water absorption and rapid chloride permeability of fly ash concrete. Constr. Build. Mater..

[47] Jang J., Kim G., Kim H., Lee H. (2016). Review on recent advances in CO_2_ utilization and sequestration technologies in cement-based materials. Constr. Build. Mater..

[48] Chen Y., Liu P., Yu Z. (2018). Effects of Environmental Factors on Concrete Carbonation Depth and Compressive Strength. Materials.

[49] Habeeb G.A., Bin Mahmud H. (2010). Study on properties of rice husk ash and its use as cement replacement material. Mater. Res..

[50] Luo M., Qian C.-X., Li R.-Y. (2015). Factors affecting crack repairing capacity of bacteria-based self-healing concrete. Constr. Build. Mater..

[51] Czarnecki L., Woyciechowski P. (2013). Prediction of the reinforced concrete structure durability under the risk of carbonation and chloride aggression. Bull. Pol. Acad. Sci. Tech. Sci..

[52] Liisma E., Sein S., Järvpõld M. (2017). The influence of carbonation process on concrete bridges and durability in Estonian practice. IOP Conf. Ser. Mater. Sci. Eng..

[53] Ghahari S.A., Ramezanianpour A.M., Esmaeili M. (2016). An Accelerated Test Method of Simultaneous Carbonation and Chloride Ion Ingress: Durability of Silica Fume Concrete in Severe Environments. Adv. Mater. Sci. Eng..

[54] Jiang S., Gao S., Jiang L., Guo M.-Z., Jiang Y., Chen C., Jin M., Bai S. (2018). Effects of Deoxyribonucleic acid on cement paste properties and chloride-induced corrosion of reinforcing steel in cement mortars. Cem. Concr. Compos..

[55] Boualleg S., Bencheikh M., Belagraa L., Daoudi A., Chikouche M.A. (2017). The Combined Effect of the Initial Cure and the Type of Cement on the Natural Carbonation, the Portlandite Content, and Nonevaporable Water in Blended Cement. Adv. Mater. Sci. Eng..

[56] Wang Y., Nanukuttan S., Bai Y., Basheer M. (2017). Influence of combined carbonation and chloride ingress regimes on rate of ingress and redistribution of chlorides in concretes. Constr. Build. Mater..

[57] Goñi S., Guerrero A. (2003). Accelerated carbonation of Friedel’s salt in calcium aluminate cement paste. Cem. Concr. Res..

[58] El-Hassan H. (2016). Carbonation Curing of Concrete Blocks to Mitigate Carbon Emission. Ph.D. Thesis.

[59] El-Hassan H. (2020). Accelerated Carbonation Curing as a Means of Reducing Carbon Dioxide Emissions. Cement Industry: Optimization, Characterization and Sustainable.

[60] El-Hassan H., Shao Y., Ghouleh Z. (2013). Reaction Products in Carbonation-Cured Lightweight Concrete. J. Mater. Civ. Eng..

[61] El-Hassan H., Shao Y. (2014). Dynamic carbonation curing of fresh lightweight concrete. Mag. Concr. Res..

[62] El-Hassan H., Shao Y. (2015). Early carbonation curing of concrete masonry units with Portland limestone cement. Cem. Concr. Compos..

[63] El-Hassan H., Shao Y. (2014). Carbon Storage through Concrete Block Carbonation. J. Clean Energy Technol..

[64] Xuan D., Zhan B., Poon C.S. (2016). Development of a new generation of eco-friendly concrete blocks by accelerated mineral carbonation. J. Clean. Prod..

[65] Lin X. (2007). Effect of Early Age Carbonation on Strength and pH of Concrete.

[66] Monkman S., Shao Y. (2010). Carbonation Curing of Slag-Cement Concrete for Binding CO_2_ and Improving Performance. J. Mater. Civ. Eng..

[67] Zhang D., Cai X., Shao Y. (2016). Carbonation Curing of Precast Fly Ash Concrete. J. Mater. Civ. Eng..

[68] Farah N., Mohamed W., Faisal A., Juki M.I. (2021). Effect of Concrete Grade on Carbonation Intrusion: A Systematic Review. Recent Trends Civ. Eng. Built Environ..

[69] Tepfers R. (2012). Concrete Technology–Porosity Is Decisive. Befestigungstechnik, Bewehrungstechnik und… II.

[70] Jin P., Wang R., Su Y., Dong H., Wang Q. (2019). Study on carbonation process of β-C2S under microbial enzymatic action. Constr. Build. Mater..

[71] Alshalif A.F., Juki M.I., Tajarudin H.A., Othman N., Al-Gheethi A.A., Shamsudin S., Altowayti W., Sabah S.A. (2022). Optimisation of self-healing of bio-foamed concrete bricks pores using Bacillus tequilensis under different temperature and CO_2_ curing conditions. Sci. Rep..

[72] Baciocchi R., Costa G., Polettini A., Pomi R. (2015). Effects of thin-film accelerated carbonation on steel slag leaching. J. Hazard. Mater..

[73] Baciocchi R., Costa G., Polettini A., Pomi R., Stramazzo A., Zingaretti D. (2016). Accelerated Carbonation of Steel Slags Using CO2 Diluted Sources: CO2 Uptakes and Energy Requirements. Front. Energy Res..

[74] Equaghebeur M., Enielsen P., Ehorckmans L., Mechelen D.E. (2015). Accelerated Carbonation of Steel Slag Compacts: Development of High-Strength Construction Materials. Front. Energy Res..

[75] Baciocchi R., Costa G., Polettini A., Pomi R. (2009). Influence of particle size on the carbonation of stainless steel slag for CO_2_ storage. Energy Procedia.

[76] Santos R.M., Van Bouwel J., Vandevelde E., Mertens G., Elsen J., Van Gerven T. (2013). Accelerated mineral carbonation of stainless steel slags for CO_2_ storage and waste valorization: Effect of process parameters on geochemical properties. Int. J. Greenh. Gas Control.

[77] Salmasi F., Mostofinejad D. (2020). Investigating the effects of bacterial activity on compressive strength and durability of natural lightweight aggregate concrete reinforced with steel fibers. Constr. Build. Mater..

[78] Wang K., Qian C., Wang R. (2016). The properties and mechanism of microbial mineralized steel slag bricks. Constr. Build. Mater..

[79] Achal V., Mukherjee A., Reddy M.S. (2011). Microbial Concrete: Way to Enhance the Durability of Building Structures. J. Mater. Civ. Eng..

[80] Wiktor V., Jonkers H. (2011). Quantification of crack-healing in novel bacteria-based self-healing concrete. Cem. Concr. Compos..

[81] Tayebani B., Mostofinejad D. (2019). Self-healing bacterial mortar with improved chloride permeability and electrical resistance. Constr. Build. Mater..

[82] Vijay K., Murmu M. (2019). Self-repairing of concrete cracks by using bacteria and basalt fiber. SN Appl. Sci..

[83] Wiktor V., Jonkers H. (2015). Field performance of bacteria-based repair system: Pilot study in a parking garage. Case Stud. Constr. Mater..

[84] Luo X., Wang J., Dooner M., Clarke J. (2015). Overview of current development in electrical energy storage technologies and the application potential in power system operation. Appl. Energy.

[85] Wang R., Jin P., Dong H., Liu Y., Ding Z., Zhang W. (2021). Effect of moist content on the bio-carbonated steel slag bricks. Constr. Build. Mater..

[86] Liu Y., Zhuge Y., Chow C.W., Keegan A., Li D., Pham P.N., Huang J., Siddique R. (2020). Properties and microstructure of concrete blocks incorporating drinking water treatment sludge exposed to early-age carbonation curing. J. Clean. Prod..

[87] Morris W.T. (1957). Research in Engineering Economy. Eng. Econ..

[88] Karimi N., Mostofinejad D. (2020). Bacillus subtilis bacteria used in fiber reinforced concrete and their effects on concrete penetrability. Constr. Build. Mater..

[89] Bisht V., Chaurasia L., Singh L.P. (2020). Studies on Corrosion and Carbonation Resistance by Bacteria-Mediated Mineralization in Concrete. ACI Mater. J..

[90] Wong L.S., Oweida A.F.M., Kong S.Y., Iqbal D.M., Regunathan P. (2020). The surface coating mechanism of polluted concrete by Candida ethanolica induced calcium carbonate mineralization. Constr. Build. Mater..

